# Calculation of the dynamic stiffness of a cantilever under torsional oscillation

**DOI:** 10.3762/bjnano.17.21

**Published:** 2026-02-13

**Authors:** Keita Nishida, Yuuki Yasui, Yoshiaki Sugimoto

**Affiliations:** 1 Department of Advanced Materials Science, The University of Tokyo, Chiba 277-8561, Japan

**Keywords:** atomic force microscopy, dynamic stiffness, energy dissipation, friction, torsional oscillation mode

## Abstract

Atomic force microscopy using Si cantilevers provides an effective means for investigating both conservative and dissipative interactions in the vertical and lateral directions between the tip and the sample. An accurate evaluation of the dynamic stiffness of the cantilever is indispensable in the quantitative analyses of the interactions. We calculated the dynamic stiffness of cantilevers under torsional oscillation based on the strain energy. Without tips, the torsional dynamic stiffness is approximately 23% larger than the static stiffness. The modification decreases to 21–23% with tips. Applying the present correction is essential for achieving quantitatively accurate stiffness values in dynamic measurements.

## Introduction

Friction serves as a fundamental mechanism of energy dissipation [1]. While friction typically arises from direct mechanical contact between surfaces, energy dissipation can also occur even in the absence of physical contact, and this dissipation is called non-contact friction [2]. Its origins have been investigated down to the nanometer scale [3–5]. In particular, the origin of non-contact friction is attributed to electromagnetic interactions between the two bodies, although its detailed mechanisms remain not fully understood [5].

Non-contact atomic force microscopy (nc-AFM) is widely employed to investigate non-contact friction through its dissipation channel. Common techniques include pendulum AFM, bimodal AFM, and quartz tuning fork AFM [6–8]. Pendulum AFM uses cantilevers with small stiffness and provides exceptional sensitivity to conservative and dissipative forces owing to the small stiffness [2,9–11]. Non-contact friction measurements with pendulum AFM on a Nb film across the superconducting transition indicate that friction is electronic in the metallic state, whereas phononic dissipation dominates in the superconducting state [10]. The cantilever in bimodal AFM oscillates in two modes, typically, the first flexural and first torsional modes [2]. The flexural mode is used for vertical tip-position control via its frequency shift, and the torsional mode detects the lateral interactions [7,12–14]. The torsional oscillation modes of AFM cantilevers are sensitive to in-plane interaction [15–17]. This method enabled highly accurate imaging of the in-plane crystalline orientation by utilizing friction information [14]. Quartz tuning fork AFM is useful as it can electrically detect energy dissipations [8]. Lateral force microscopy with quartz tuning fork AFM, using a CO-terminated tip, enabled the detection of energy dissipation over the chemical bonds of a PTCDA (3,4,9,10-perylenetetracarboxylic dianhydride) molecule, with a vertical decay length of 4 pm [18].

Quantitative interpretation of the conservative interaction energy and the energy dissipation requires the stiffness of the oscillators [19–21]. The oscillator exhibits different stiffnesses in dynamic and static cases [22–24]. While the static stiffness is easily obtained from the geometrical structure [2], an accurate evaluation of dynamic stiffness requires detailed analyses of the oscillator dynamics. The modification from static to dynamic stiffness depends on the oscillation mode because each mode is governed by a different equation of motion reflecting the underlying deformation mechanism.

Here, we calculated the dynamic stiffness of cantilevers with tips in torsional oscillation using the equivalent spring-moment of inertia model. Then we found that the dynamic stiffness should be modified by 21–23% when the tip is considered. Dissipated energy is derived from the excitation amplitude through a proportionality coefficient that depends on the dynamic stiffness [21]. Thus, the present correction for the dynamic stiffness should be adopted for friction analyses with torsional oscillations in nc-AFM.

## Model

**Figure 1:**
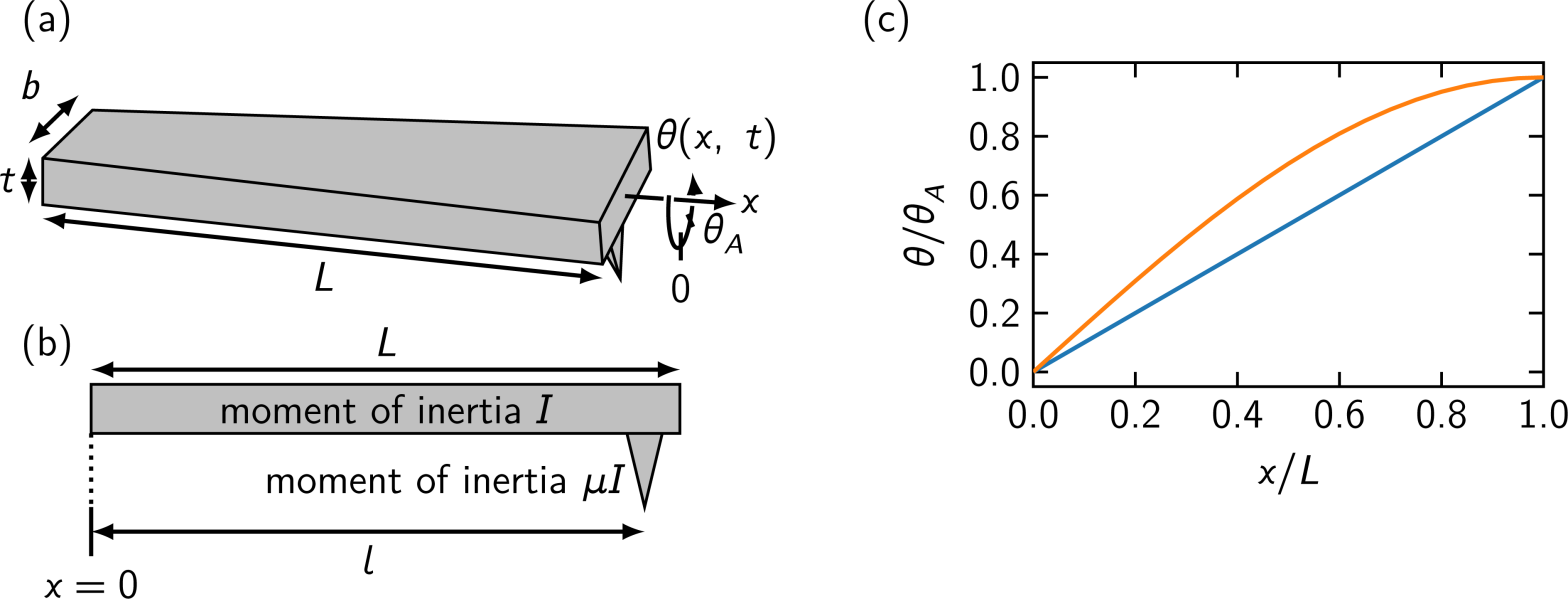
(a) Schematic image of a model cantilever. (b) Side view of the cantilever. A tip with a moment of inertia μ*I* is at *x* = *l*. (c) Displacement in the torsional direction for the static deformation (blue) and the dynamic deformation in fundamental mode (orange) for μ = 0 and *l* = *L*.

We consider a cantilever with the dimensions length *L*, width *b*, and thickness *t* with *L*, *b* ≫ *t* as shown in Figure 1a. The corresponding moment of inertia along the *x* axis is *I* = ρ*b*^3^*tL*/12, where ρ is the mass density and is uniform in the cantilever. The angle of the torsional displacement is denoted by θ(*x*, *t*) at the position *x* and the time *t*, and the angle for the oscillation amplitude is represented by θ*_A_*. The cantilever is fixed at *x* = 0 and is free at *x* = *L*. A tip is attached at *x* = *l*, and the moment of inertia of the tip along the *x* axis is μ*I* with μ ≪ 1, as illustrated in Figure 1b. Note that the geometric contribution of the tip is included in the deduced moment of inertia.

The oscillator is modelled as an equivalent spring-moment of inertia model [22,24], and the static stiffness and the dynamic stiffness under torsional oscillation are calculated. The equation of motion of the oscillator under the torsional oscillation is given by


[1] $$\rho I_{\text{p}}\frac{\partial^{2}\theta }{\partial t^{2}}=C_{\text{t}}\frac{\partial^{2}\theta }{\partial x^{2}},$$

where *I*_p_ = *b*^3^*t*/12 is the polar moment of inertia, and *C*_t_ is the torsional rigidity [25–26].

## Results

First, we consider a static torsion. The solution to Equation 1 is


[2] $$\theta_{0}\left(x,\;t\right)=\theta_{A}\frac{x}{l}$$

under the boundary conditions of θ(0, *t*) = 0 and θ(*l*, *t*) = θ*_A_*.

Next, we consider dynamic oscillations with a specific tip configuration, where the tip has a negligible moment of inertia (μ = 0) and is at *x* = *L*. The boundary conditions are θ(0, *t*) = 0 (no torsion) and *C*_t_θ′(*L*, *t*) = 0 (no torque) [27]. With these conditions, we obtain


[3] $$\theta_{n}\left(x,\;t\right)=\theta_{A}\sin\left(\frac{2n-1}{2L}\pi x\right)\cos\left(\omega_{n}t\right),$$

where 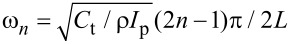 is the resonance angular frequency for the *n*-th eigenmode [28–29]. Figure 1c shows the deformation of the cantilever for the fundamental oscillation mode θ_1_(*x*, 0) and for the static displacement θ_0_(*x*, 0). In the dynamic case, the local torsion is concentrated to the range near the fixed end of the cantilever compared with the static case.

We then treat the torsional oscillations using the spring-moment of inertia model and derive the formula for the dynamic stiffness by assuming that the kinetic energy and the strain energy of the cantilever are equivalent to those of the model [22,30]. Specifically, for the kinetic energy 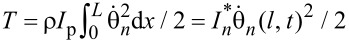, and for the strain energy 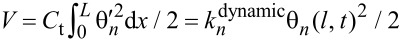, where 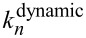 is the dynamic stiffness for the *n*-th oscillation mode, and 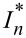 is the effective moment of inertia. The dynamic stiffness and the effective moment of inertia are then given by


[4] $$k_{n}^{\text{dynamic}}=\frac{k^{\text{static}}}{2}{\left(\frac{2n-1}{2}\pi \right)}^{2},$$

[5] $$I_{n}^{*}=\frac{I}{2},$$

where *k*^static^ is the static stiffness defined by *k*^static^ = *C*_t_/*L*. The resonance angular frequencies obtained as 
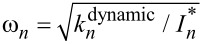
 are equal to those defined for Equation 3. Dynamic stiffness differs from static stiffness for all modes. For the fundamental mode, 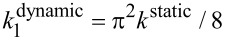, and thus the dynamic stiffness is approximately 23% larger than the static stiffness. The effective moment of inertia is equal to half the moment of inertia of the cantilever for any oscillation modes.

Now, more general configurations are considered, where the tip is at an arbitrary position and has a finite moment of inertia. The boundary conditions should be modified as both the position and the moment of inertia of the tip affect the conditions. The cantilever is divided into two parts; the deformation is described as θ_left_(*x*, *t*) for 0 ≤ *x* ≤ *l* and θ_right_(*x*, *t*) for *l < x* ≤ *L*. The boundary conditions [25] are


[6]

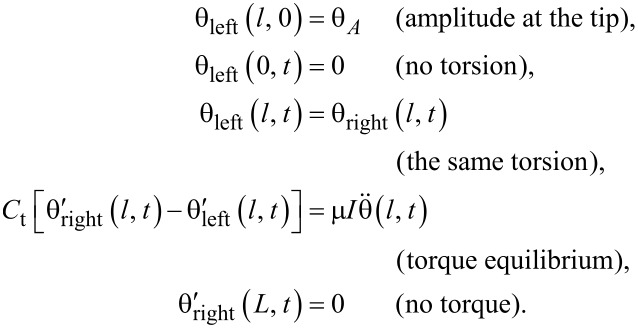



The equation of motion yields


[7]

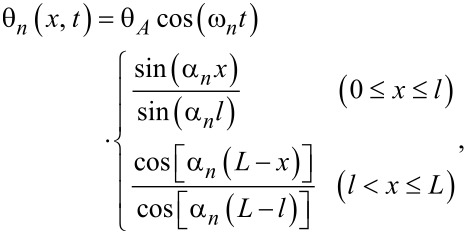



where the parameters α*_n_*(*l*, μ) are the positive solutions of


[8] $$\mu \alpha_{n}L\sin\left(\alpha_{n}l\right)\cos\left[\alpha_{n}\left(L-l\right)\right]-\cos\left(\alpha_{n}L\right)=0$$

in ascending order, and 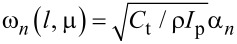 is the resonance angular frequency for the *n*-th eigenmode. The resonance angular frequency becomes lower when a tip with larger moment of inertia is attached, consistent with previous research [31].

**Figure 2:**
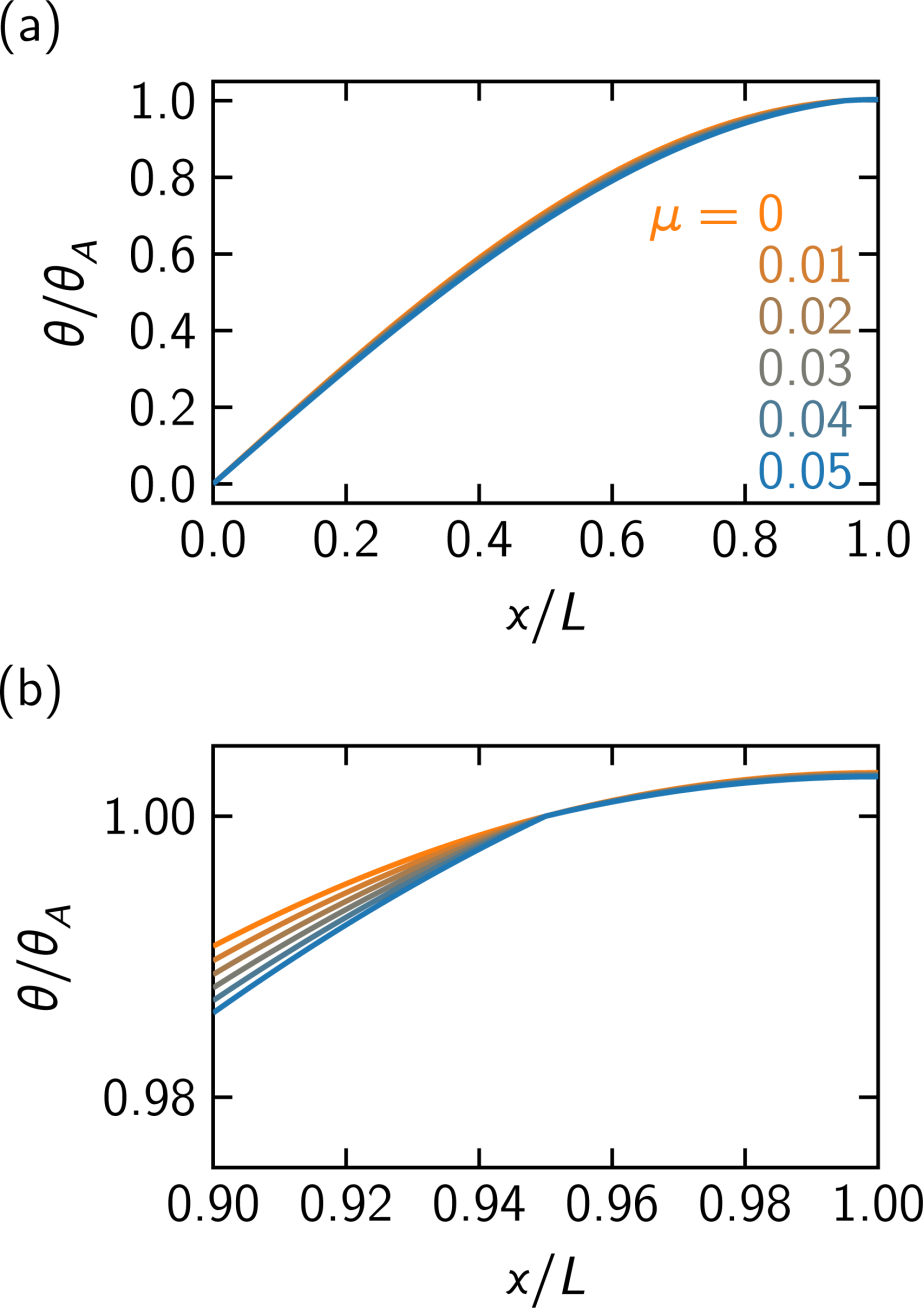
The effect of the moment of inertia of the tip. The deformation of cantilevers under the fundamental mode are illustrated. The tip is attached at *l*/*L* = 0.95. (a) Overall curves and (b) zoomed-in view of (a).

**Figure 3:**
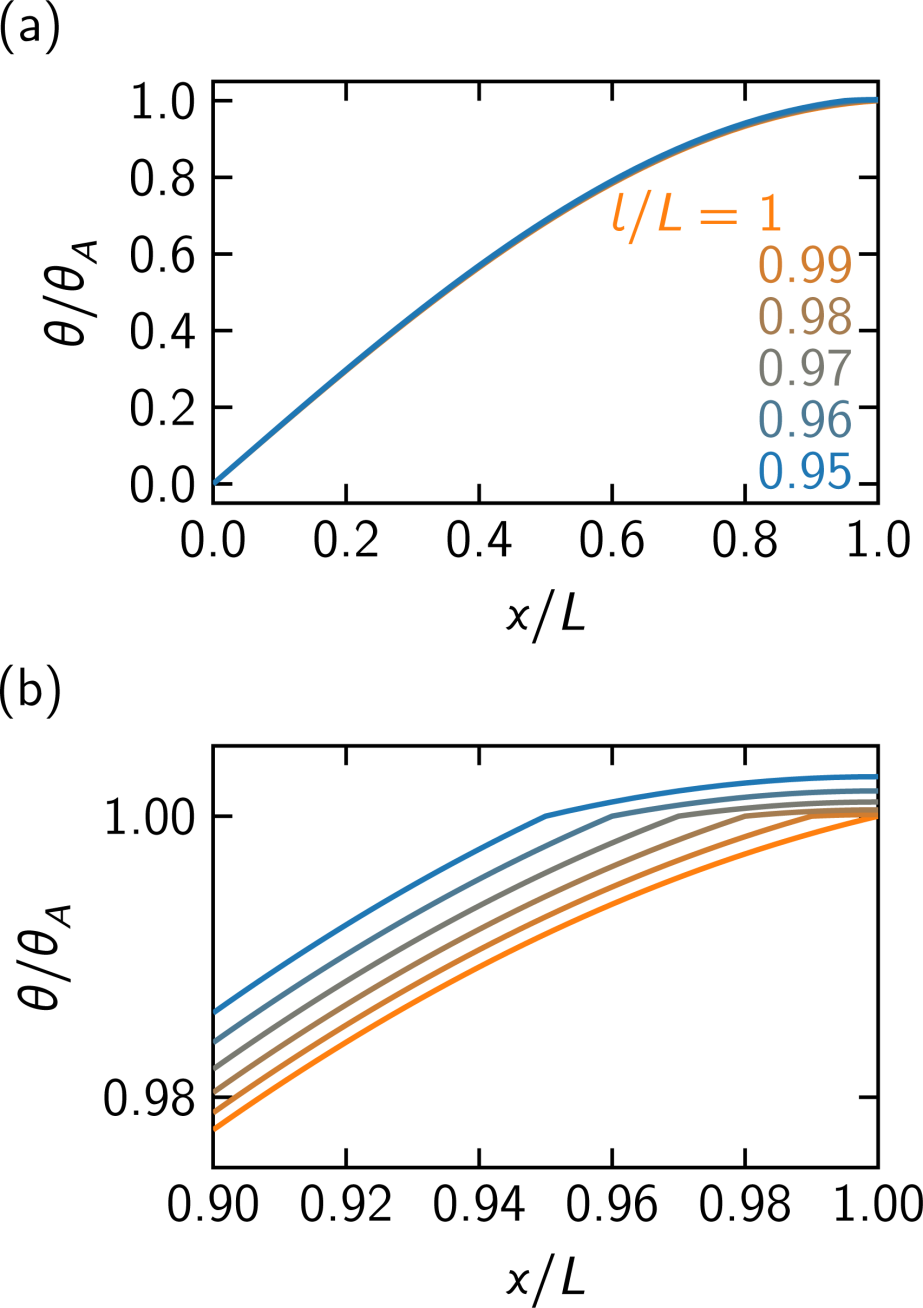
The effect of the moment of inertia of the tip. The deformation of cantilevers under the fundamental mode are depicted. The moment of inertia of the tip is μ = 0.05. (a) Overall curves and (b) zoomed-in view of (a).

Figure 2 illustrates the deformation of the cantilevers with tips with various moments of inertia at *l*/*L* = 0.95 in the fundamental oscillation mode. As the tip with larger moment of inertia lowers the resonance angular frequency, the cantilever torsion becomes similar to the situation in the static case. The gradient of the deformation for *l < x* ≤ *L* is smaller than that of the displacement for 0 ≤ *x* ≤ *l* due to the effect of the tip as shown in the boundary conditions in Equation 6. Figure 3 shows the displacement of the cantilever with a tip of a moment of inertia μ = 0.05 at various positions under the fundamental mode. When the tip is attached closer to the inner part of the cantilever, the local torsion of the cantilever becomes larger for 0 ≤ *x* ≤ *l* to maintain the amplitude at *x* = *l*, and the gradients of the deformation become smaller for *l < x* ≤ *L*.

The dynamic stiffness and the effective moment of inertia with a tip are also calculated. The kinetic energy is now modified to be 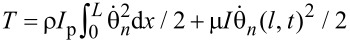, while the strain energy 
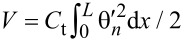
 remains the same [22,30]. The dynamic stiffness 
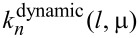
 and the effective moment of inertia 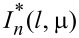 are derived as


[9] $$k_{n}^{\text{dynamic}}\left(l,\;\mu \right)=\frac{k^{\text{static}}}{2}{\left(\alpha_{n}L\right)}^{2}\frac{P\left(l,\;\mu \right)}{Q\left(l,\;\mu \right)},$$

[10] $$I_{n}^{*}\left(l,\;\mu \right)=\frac{I}{2}\frac{P\left(l,\;\mu \right)}{Q\left(l,\;\mu \right)},$$

where *P*(*l*, μ) and *Q*(*l*, μ) are


[11] $$\begin{array}{c} P\left(l,\;\mu \right)=\alpha_{n}L\sin^{2}\left(\alpha_{n}l\right)\\ +\alpha_{n}l\cos\left(\alpha_{n}L\right)\cos\left[\alpha_{n}\left(L-2l\right)\right]\\ +\cos\left(\alpha_{n}L\right)\sin\left(\alpha_{n}l\right)\cos\left[\alpha_{n}(L-l)\right], \end{array}$$

[12] $$Q\left(l,\;\mu \right)=\alpha_{n}L\sin^{2}\left(\alpha_{n}l\right)\cos^{2}\left[\alpha_{n}\left(L-l\right)\right].$$

**Figure 4:**
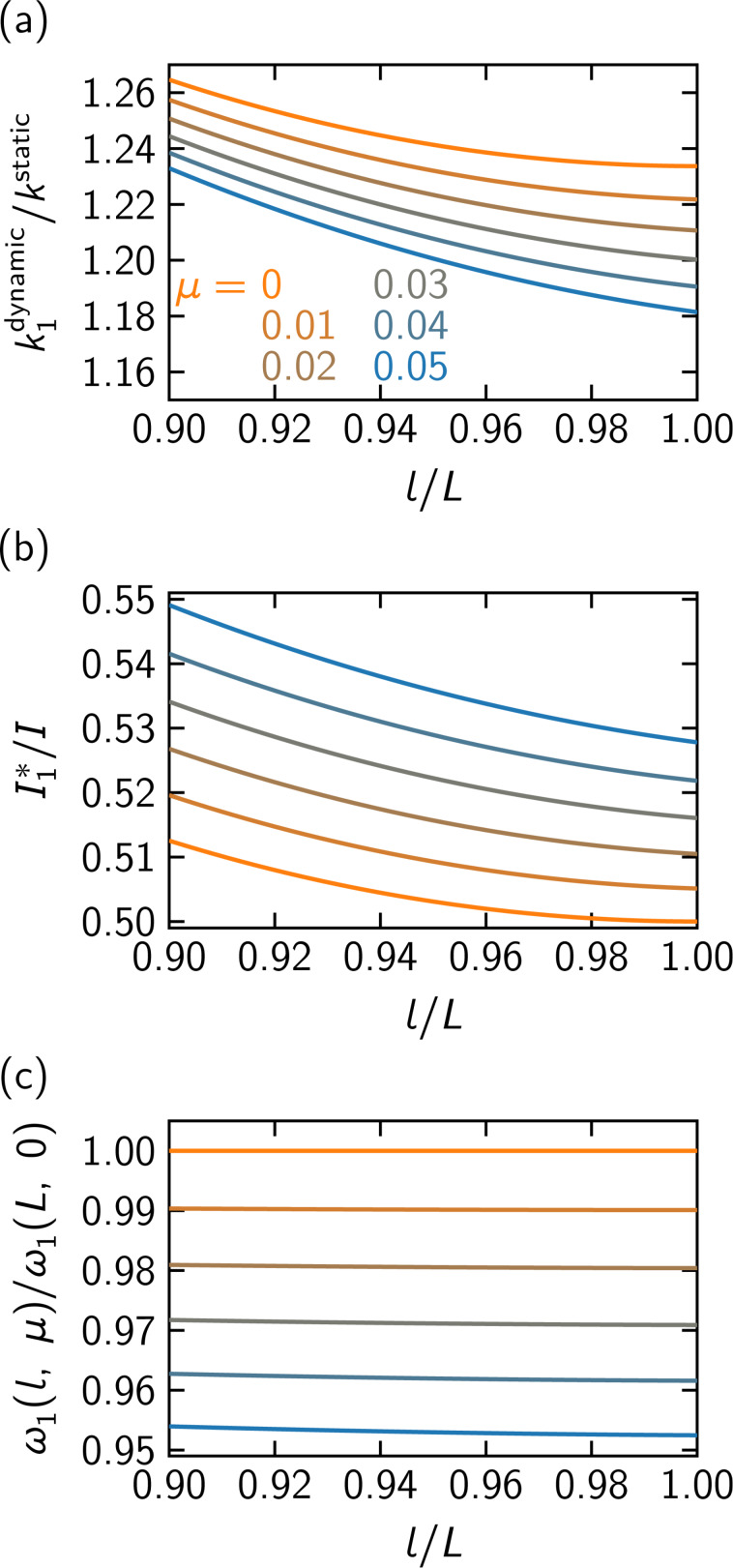
(a) The dynamic stiffness, (b) the effective moment of inertia, and (c) the fundamental resonance angular frequency when a tip of a moment of inertia μ*I* is attached to the cantilever at *x* = *l*.

The dynamic stiffness, the effective moment of inertia, and the resonance angular frequency in the fundamental oscillation mode are plotted in Figure 4. As μ increases, the dynamic stiffness decreases. In contrast, the dynamic stiffness increases as the tip is positioned away from the cantilever end since this configuration leads to larger local torsion of the cantilever. The effective moment of inertia is influenced by the oscillation mode when the tip is attached. It increases as the tip with larger moment of inertia is positioned closer to the inner part of the cantilever. The resonance angular frequencies calculated with 
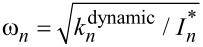
 are consistent with the values defined for Equation 7. For the fundamental mode, the resonance frequency is only minimally affected by attaching the tip with small moment of inertia near the free end of the cantilever. It becomes lower with the tip with larger moment of inertia, while it grows higher when the tip is positioned away from the free end of the cantilever.

## Discussion

To know the practical effect of the tip to the oscillating cantilever, we consider two specific cantilever configurations, namely, a long cantilever and a short cantilever. The cantilever geometries are shown in Table 1. The tips in the configurations are assumed to have the same cone shape with the height *h* = 17 μm and the base radius *r* = 8 μm and to be positioned at *l*/*L* = 0.97 and *l*/*L* = 0.94 for the long cantilever and the short cantilever, respectively. The ratio of the dynamic stiffness to the static stiffness for the fundamental mode is also shown in Table 1. The dynamic stiffness is larger by 23% for the long cantilever and by 21% for the short cantilever. This implies that the non-contact friction is underestimated by around 20% if the present correction is not adopted.

**Table 1:** Dynamic stiffness for two cantilever configurations.

	long cantilever	short cantilever

*L* [μm]	225	125
*b* [μm]	38	30
*t* [μm]	7	4
*l*/*L*	0.97	0.94
μ	0.008	0.043
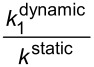	1.23	1.21

In conclusion, we derived formulae for the dynamic stiffness of the cantilever in torsional oscillation through a comparison of the oscillating cantilever and the equivalent spring-moment of inertia model. The dynamic stiffness is 21% and 23% larger than the static stiffness for specific configurations of cantilevers and tips, implying the importance of the present corrections for accurate measurements of non-contact friction. The present modification can be experimentally evaluated by comparing the lateral force measured with the torsional oscillation mode and that from three-dimensional force maps obtained using vertical oscillation.

## Data Availability

Data generated and analyzed during this study is available from the corresponding author upon reasonable request.
